# Characterization of Atrial and Ventricular Structural Remodeling in a Porcine Model of Atrial Fibrillation Induced by Atrial Tachypacing

**DOI:** 10.3389/fvets.2020.00179

**Published:** 2020-04-09

**Authors:** Carlotta Citerni, Jeppe Kirchhoff, Lisbeth Høier Olsen, Stefan Michael Sattler, Fabio Gentilini, Monica Forni, Augusta Zannoni, Morten Grunnet, Nils Edvardsson, Bo Hjorth Bentzen, Jonas Goldin Diness

**Affiliations:** ^1^Biomedical Institute, University of Copenhagen, Copenhagen, Denmark; ^2^Acesion Pharma, Copenhagen, Denmark; ^3^Department of Veterinary and Animal Sciences, University of Copenhagen, Frederiksberg, Denmark; ^4^Department of Cardiology, Heart Center, Copenhagen University Hospital, Rigshospitalet, Copenhagen, Denmark; ^5^Medical Department I, University Hospital Grosshadern, LMU Munich, Munich, Germany; ^6^Department of Veterinary Medical Sciences, University of Bologna, Bologna, Italy; ^7^Department of Molecular and Clinical Medicine/Cardiology, Institute of Medicine, Sahlgrenska Academy, University of Gothenburg, Gothenburg, Sweden

**Keywords:** left ventricular dysfunction, atrial fibrillation, pig model, remodeling, echocardiography

## Abstract

**Background:** Atrial fibrillation (AF) is characterized by electrical and structural remodeling. Irregular and/or fast atrio-ventricular (AV) conduction during AF can result in AV dyssynchrony, tachymyopathy, pressure and volume overload with subsequent dilatation, valve regurgitation, and ventricular dysfunction with progression to heart failure.

**Objective:** To gain further insight into the myocardial pathophysiological changes induced by right atrial tachypacing (A-TP) in a large animal model.

**Methods:** A total of 28 Landrace pigs were randomized as 14 into AF-induced A-TP group and 14 pigs to control group. AF pigs were tachypaced for 43 ± 4 days until in sustained AF. Functional remodeling was investigated by echocardiography (after cardioversion to sinus rhythm). Structural remodeling was quantified by histological preparations with picrosirius red and immunohistochemical stainings.

**Results:** A-TP resulted in decreased left ventricular ejection fraction (LVEF) accompanied by increased end-diastolic and end-systolic left atrium (LA) volume and area. In addition, A-TP was associated with mitral valve (MV) regurgitation, diastolic dysfunction and increased atrial and ventricular fibrotic extracellular matrix (ECM).

**Conclusions:** A-TP induced AF with concomitant LV systolic and diastolic dysfunction, increased LA volume and area, and atrial and ventricular fibrosis.

## Introduction

Atrial fibrillation, left ventricular dysfunction and clinical heart failure are interrelated conditions promoting each other and both are associated with increased mortality.

Atrial fibrillation promotes atrial and ventricular remodeling by acute and chronic hemodynamic effects. Diastolic filling is impaired due to rapid ventricular rate and irregular rhythm reducing the interval for passive diastolic filling. The loss of atrial contribution to ventricular filling reduces end-diastolic pressure and volume in both ventricles (1–5). Atrial fibrillation has also been suggested to cause functional mitral valve regurgitation often secondary to left atrial and annular enlargement and left ventricular remodeling (6–8). A chronic fast and/or irregular atrio-ventricular conduction can lead to tachycardia-mediated cardiomyopathy which in turn can cause or worsen left ventricular dysfunction and/or heart failure.

We have recently developed a porcine model with atrial tachypacing (A-TP) induced atrial fibrillation that was treatment-resistant to vernakalant (9). We expected that pigs with treatment-resistant atrial fibrillation will develop signs of ventricular dysfunction, such as a reduced left ventricular ejection fraction (LVEF), increased fibrosis and enlarged atrial size. If that is the case, we hope to use this model to test therapies for atrial fibrillation in a context of myocardial remodeling and/or heart failure. The aim of this study was to explore early signs of functional and structural myocardial remodeling by means of echocardiography and post-mortem histological analyses in a large animal porcine model.

## Materials and Methods

All animal studies were performed under a license from the Danish Ministry of Environment and Food (license No. 2014-15-0201-00390), in accordance with the Danish guidelines for animal experiments according to the European Commission Directive 86/609/EEC. For details on materials used in this study, please refer to the Supplementary Materials section.

### Implantation of Leads and Pacemakers/Neurostimulators in Pigs

A total of 28 Danish landrace pigs (gilts) had cardiac pacing devices implanted at an age of 11 weeks (25–35 kg). Pigs were premedicated with zoletil pig mixture and intubated and ventilated with a tidal volume of 10 ml/kg and a respiration frequency of 12–14/min. Anesthesia was maintained with propofol and fentanyl (15 mg/kg/h and 5 μg/kg/h, respectively). During surgery paCO_2_, blood pressure and electrocardiogram (ECG) were monitored and the pig was given 6 ml/kg/h isotonic saline. Under aseptic conditions and fluoroscopic guidance, one or two bipolar pacing-electrode leads were inserted into the right atrium and connected to a pacing device implanted in the neck.

**Figure 1:**
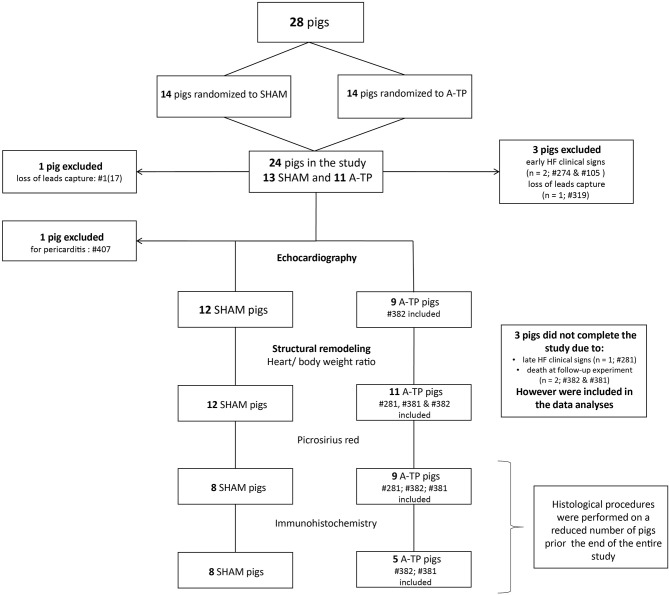
Flowchart of the pigs randomized into A-TP and SHAM group with included and excluded animals within the single procedures of the study.

The pigs were divided into two groups: one group intended for long-term A-TP and atrial fibrillation induction and a control group of sham operated pigs (SHAM) (See Figure 1 and Supplementary Materials for more details). In the A-TP pigs one electrode was implanted in the right atrium and connected to a neurostimulator. In the SHAM pigs two electrodes were placed in the same position and connected to a pacemaker with diagnostic functions.

### Pacing Protocol

The A-TP pigs were tachypaced at a constant rate of 420 impulses/min until development of sustained AF that was resistant to cardioversion by the atrial selective antiarrhythmic drug vernakalant (4 mg/kg) as described in (9). On average, this took 18 ± 2 days of A-TP. The pigs were then paced for additionally 25 ± 4 days for a total of 43 ± 4 days of A-TP before follow-up. In patients, the efficacy of vernakalant declines with increasing atrial fibrillation duration and therefore resistance to vernakalant-treatment was used in this study as a marker of atrial fibrillation-induced remodeling. All pigs were treated with a daily dose of 250 μg digoxin in order to depress AV conduction, decrease the ventricular rate, and prevent or slow the development of clinical signs of heart failure (9).

### Echocardiography

At follow-up after 43 ± 4 days of A-TP, the pigs were anaesthetized and catheterized for measurements of blood pressure through the right femoral artery. Biphasic direct current cardioversion with 250 joules was performed on the pigs with AF 60 ± 20 min after induction of anesthesia.

**Figure 2:**
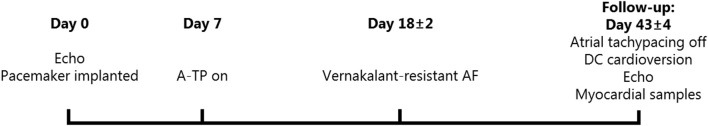
Timeline of the study with indication of the actions taken at each time point. SHAM pigs have a comparable flowchart, except for A-TP and development of atrial fibrillation. A-TP, atrial tachypacing; AF, atrial fibrillation; DC, direct current.

**Figure 3:**
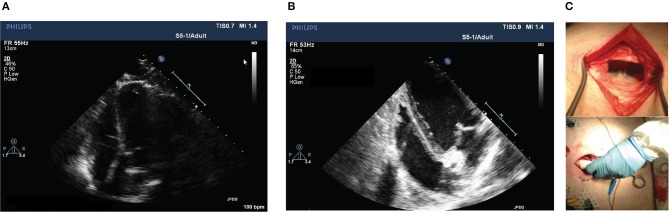
Comparison between echocardiographic techniques in the substernal window. **(A)** Standard trans-thoracic echocardiography (TTE) and **(B)** Newly introduced trans-diaphragm echocardiography (TDE). Images of apical 4-chamber view of the porcine heart are slightly rotated but quality improved with TDE technique. **(C)** Approach to TDE creating a surgical window under the xiphoid process and a preparation toward the diaphragm (top image) allows placing the probe directly on the diaphragm (bottom).

Acquisition of images was performed using an iE33 machine (Philips Health care, Amsterdam, The Netherlands) equipped with a S5-1 transducer (3.5 MHz). Echocardiograms were obtained during anesthesia on the day of pacemaker implantation and at follow-up 30 ± 10 min after direct current (DC) cardioversion (Figure 2). Standardized images were obtained following the recommendations for transthoracic echocardiography (TTE) in left and right lateral recumbency when possible (10, 11). Additionally, echocardiography in dorsal recumbency was included. All examinations were done by the same operator. Using right parasternal transducer location, short-axis (SAX) views at the chordae tendinae of the left ventricle were obtained. From left apical transducer location, apical 4-chamber and 5-chamber views were obtained. Due to the size of the pigs at the time of the terminal follow-up (68.8 ± 4.3 kg for A-TP and 69.4 ± 3.3 kg for SHAM pigs), a surgical window under the xiphoid process (sub sternum) was made in order to place the probe directly on the diaphragm. Slightly rotated but higher quality images of apical views were obtained with the pig in dorsal recumbency. This trans-diaphragm echocardiography (TDE) technique is described in Figure 3 and in greater detail in the Supplementary Materials as well as in (12). All echocardiographic data were analyzed in a blinded fashion. All reported values are averages of three sequential heart cycles in sinus rhythm. Left ventricular systolic function was evaluated by using two-dimensional guided M-mode performed on the SAX view and fractional shortening (FS) using left ventricular internal diameters in systole and diastole (11). In addition, maximum diastolic and systolic left ventricular internal areas were measured in SAX and used to calculate the fraction area change (FAC) (10, 11). Finally, LVEF was estimated using Simpson's mode (SMOD) including, estimations of maximum diastolic and systolic left ventricular volumes from the left apical 4-chamber view. Using the same view, left atrial volume was evaluated with the biplane modified Simpson's method of disks and the area with the maximal left atrial circumference before mitral valve opening. Mitral valve (MV) regurgitation was evaluated in the apical 4-chamber view. When present, the mitral regurgitation was defined as minimal (≤ 10% of the left atrial surface), mild (>10 and ≤50% of left atrial surface) or moderate to severe (>50% of the left atrial surface) (10). Valvular insufficiencies were classified referring to existing literature (10). Peak aorta flow velocity and presence of aortic regurgitation were evaluated in the apical 5-chamber view. As an estimation of right ventricular function, tricuspid annular plane systolic excursion (TAPSE) was measured (13). Diastolic function was investigated by evaluation of MV inflow Doppler patterns using peak E (early diastole), peak A (late diastole), transmitral E/A ratio and E velocity deceleration time (EDT) to identify the filling patterns and myocardial stiffness. Tissue Doppler Imaging using estimations of septal (e' septal) and lateral free wall (e' lateral) myocardial velocities to calculate the velocity of the mitral annulus early diastolic wave (septal and lateral E/e' ratio) can be used to predict left ventricular filling pressures and relaxation (14, 15).

### Analysis of Structural Remodeling and Fibrosis

Structural remodeling was investigated histologically by picrosirius red stainings and by lectin histochemistry and vimentin immunohistochemistry.

#### Tissue Samples

Cardiac tissue samples from all four cardiac chambers were obtained at the end of the study. The hearts were explanted and immediately placed in cardioplegic solution (see Supplementary Materials), weighed and sampled for immunohistochemistry and histological analysis. The total weights of the heart and each of the chambers were normalized to body weight, which had increased from 31.7 ± 0.6 kg for A-TP and 32.4 ± 0.6 kg for SHAM pigs at baseline to 68.8 ± 4.3 and 69.4 ± 3.3 kg, respectively, at follow-up 43 ± 4 days later.

#### Picrosirius Red Staining for Collagen

**Figure 4:**
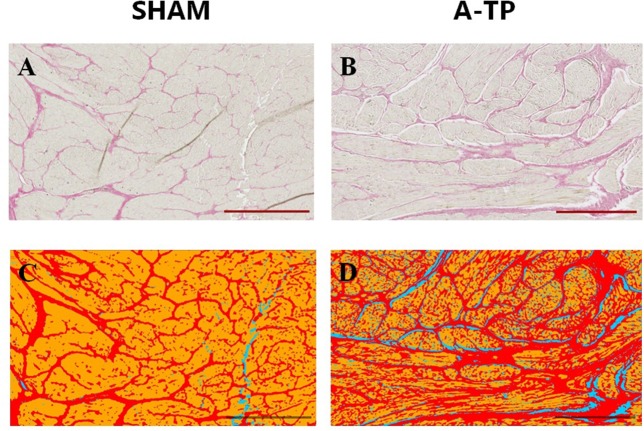
Picrosirius red staining for collagen in the left atrial tissue of pigs. Scale bars indicate 500 μm. Picrosirius red stains the collagen in red and the cardiomyocytes in yellow **(A,B)**. The trained program Intellesis Zen 2.3 recognizes the fibrotic tissue in red, the background in blue and the cardiomyocytes in yellow **(C,D)**.

Tissue samples from the free wall (1 × 1 cm, avoiding areas where leads were implanted) of left atrium, right atrium, left ventricle and right ventricle from 8 SHAM pigs and 9 A-TP pigs were used for picrosirius red staining to separate and quantify the relative amounts of cardiomyocytes, collagen and background. Each location was sliced in a maximum of three selections (4 μm). Slides were automatically scanned by the confocal microscope Axio Scan.Z1 slide scanner (Zeiss, Germany) with 20X magnification with 0.8 objective for brightfield lens. From each slide, 3- 12 regions of interest (ROI) were randomly selected and blindly scored by an unrelated person aiming to cover between 10 and 15% of each selection for a total of 30–45% of each location. From 3 to 20 ROIs per chamber resulting in a total of 22–50 images per animal were collected and analyzed. The selection criteria aimed to exclude epicardium, endocardial adipose tissue and large blood vessels from the chosen ROI (Figures 4A,B). Image segmentation was done by creating a trained model (Figures 4C,D) in order to differentiate between cardiomyocytes, collagen and background (ZEN Intellesis, Zeiss, Germany). The analyses were performed with ZEN 2.3 Blue edition software (Zeiss, Germany). A detailed description of the trained model can be found in the Supplementary Materials.

#### Lectin Histochemistry and Vimentin Immunohistochemistry

Tissue samples from the free wall (1 × 1 cm, avoiding areas where leads were implanted) of left atrium, right atrium, left ventricle and right ventricle from 8 SHAM pigs and 5 A-TP pigs were used for lectin histochemistry and vimentin immunohistochemistry. Tissue was cryosectioned (8 μm), mounted on glass slides and stored at −80°C. In order to separate and quantify the relative areas of extracellular matrix, capillaries, fibroblasts, and cardiomyocytes, the slides were stained with a triple immunofluorescence staining (WGA - GSIB4 - vimentin antibody with goat anti-mouse IgG). Wheat Germ Agglutinin (WGA, Alexa Fluor™ 647 Conjugate, Invitrogen, Thermo Fisher Scientific, Landsmeer, The Netherlands) is a lectin used to stain the connective tissue by binding to glycoproteins and proteoglycans (16). Isolectin B4 (GS-IB4, from Griffonia simplicifolia, Alexa Fluor™ 488 Conjugate, Invitrogen, Thermo Fisher Scientific, Landsmeer, The Netherlands) ligates to residues expressed on the surface of endothelial cells staining capillaries (17). Vimentin antibody with its secondary antibody (Goat Anti-Mouse IgG H&L, Abcam, Cambridge, United Kingdom) were used for fibroblast staining. Vimentin antibodies bond to the cytoskeletal structure of vimentin thereby marking fibroblasts (18). WGA and GS-IB4 were diluted at 1/100, primary vimentin antibody at 1/50, and secondary goat anti-mouse IgG antibody at 1/200 with a blocking solution containing bovine serum albumin (Albumin Fraction V fatty acid-free, Roche Diagnostics GmbH, Germany) and glycine (Glycine ≥ 99%, Sigma-Aldrich, Germany) to prevent non-specific binding of antibodies. Slides were visualized by confocal fluorescence microscopy using a Leica DM4B microscope combined with a Leica MC170HD camera and LAS v 4.8 imaging software (Leica, Wetzlar, Germany). Images for further analysis were captured at 400X magnification and 3–4 fields of view per location, filtered for emissions of different wavelengths. Twelve to thirteen images/location/stain/animal were collected and analyzed. Only in one animal one field of view for three locations (left atrium, right atrium, and right ventricle) was excluded from the analysis for poor image quality, ending up with the number of two fields of view per location.

**Figure 5:**
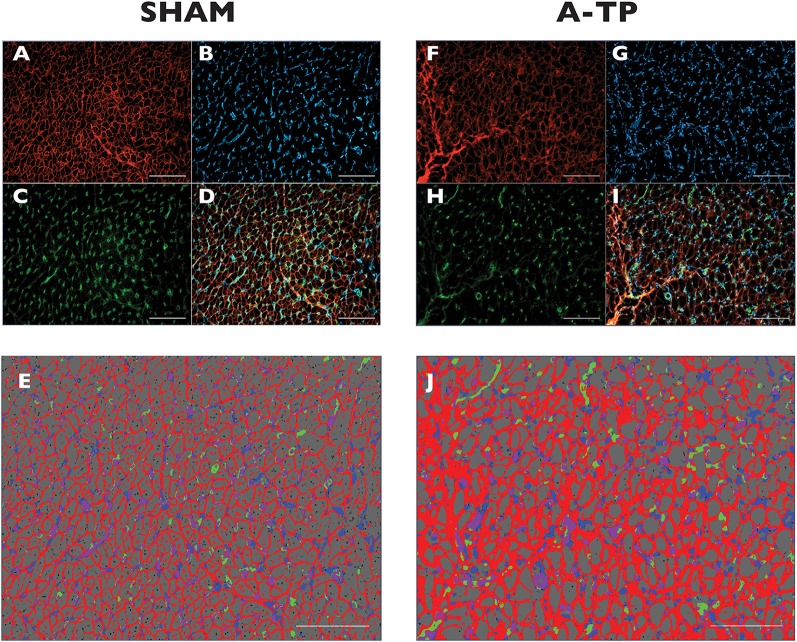
Immunofluorescence staining for extracellular matrix (ECM) in the left atrial tissue of pigs. Original magnification is 400 x. Scale bars indicate 500 μm. The triple immunofluorescence stained the WGA-expressing tissue in red **(A,F)**, the vimentin-expressing cells (fibroblasts) in blue **(B,G)** and the Isolectin B4 (GSI-B4)-sensitive capillaries in green **(C,H)**. On the merged ROIs **(D,I)** the Intellesis software recognized ECM (red), background (grey), fibroblasts (blue), capillaries (green) and the overlapping of capillaries and fibroblasts (purple) **(E,J)**.

For analysis, the filter channels were split (Figures 5A–C,F–H) using Image J Fiji (online open source platform) and merged (Figures 5D,I) with Zen 2.5 Blue Edition (Zeiss, DE). The merged images were selected for segmentation by creating a trained model with the Intellesis program system in ZEN 2.5. The model was trained to separate the fibrotic extracellular matrix, cardiomyocytes, the capillaries, the fibroblasts, and the overlapping of these two latter (Figures 5E,J). Data results from each cardiac chamber are an average of the selected fields of view.

### Statistical Analyses

GraphPad Prism 8 was used for statistical analyses. Unless otherwise indicated all data sets were assumed to come from a Gaussian distribution and analyzed with unpaired Student's t-tests with Holm-Sidak's correction for multiple comparisons. Each row was analyzed individually, without assuming a consistent SD. The number of pigs with mitral regurgitation was analyzed with Fisher's exact test comparing the incidence of no, minimal or mild to severe mitral regurgitation in the two groups. In figures, *P*-values < 0.05 are given with three decimals. In tables, all *P*-values are given with three decimals. *P*-values < 0.05 were considered statistically significant.

## Results

Out of a total of 28 pigs, 24 pigs were included in the study (Figure 1). Two A-TP pigs were euthanized due to early clinical signs of heart failure and two pigs for loss of pacemaker lead capture. One A-TP pig was euthanized due to late signs of heart failure few days before the follow-up experiment. However, the latter was included in the cardiac tissue analyses. One SHAM pig was excluded due to pericarditis. A total of 23 pigs (12 SHAM and 11 A-TP) were included in the heart weight/body weight ratio data analysis. Two A-TP pigs died during the follow-up experiment. The remaining 21 pigs (12 SHAM and 9 A-TP pigs) without clinical signs of heart failure were subjected to echocardiographic analyses. The histological procedures were performed on tissue from a reduced number of pigs (see the flowchart in Figure 1). Tissue from 17 pigs (9 A-TP and 8 SHAM pigs) was used for picrosirius red staining. Tissue from 13 pigs (5 A-TP and 8 SHAM) was used for the immunohistochemical analysis. No significant difference in body weight between the groups was observed at baseline or at follow-up (Table 1). However, as expected, the heart rate in A-TP pigs during atrial fibrillation was significantly higher than in SHAM pigs at follow up (137 ± 12 bpm for A-TP pigs in atrial fibrillation vs. 74 ± 4 bpm for SHAM pigs, *p* < 0.001).

**Table 1:** Echocardiographic evaluation at follow-up.

	**SHAM follow up *n* = 12**	**A-TP follow up (SR) *n* = 9**	***P*-value**
Body weight (kg)	69 ± 4	69 ± 4	0.959
Heart rate (bpm)	74 ± 4	80 ± 6	0.504
Left ventricular internal diameter in diastole (mm)	47 ± 1	47 ± 4^7^	0.870
Left ventricular internal diameter in systole (mm)	26 ± 1	29 ± 3^7^	0.673
Fractional Shortening; FS (%)	45 ± 2	48 ± 4	0.673
LV end-diastolic Vol. (ml)	106 ± 3^11^	131 ± 9	0.054
LV end-systolic Vol. (ml)	34 ± 3^11^	63 ± 5	<0.001
Ejection Fraction; EF (%)	68 ± 2^11^	48 ± 4	0.001
LV Fractional Area Change; FAC (%)	63 ± 2	41 ± 4^7^	<0.001
Peak aortic flow velocity (cm/s)	1.3 ± 0.1^11^	1.1 ± 0.1^8^	0.223

*The A-TP were cardioverted and in sinus rhythm for 30 min before echocardiography (SR). Volumes of LA and LV and LA area measured with SMOD method in left apical 4 chamber view; LV area in systole and diastole measured in short-axis view using two-dimensional echocardiography. Superscripts are the number of animals from which the average in question has been calculated in case of missing observations. Values are expressed as mean ± SEM. Adjusted *P*-values are expressed in the right column (multiple Student t-tests with Holm-Sidak's correction for multiple comparisons. Each row was analyzed individually, without assuming a consistent SD. Mann-Whitney test for body weight and HR)*.

### Echocardiography and Heart Weight

**Figure 6:**
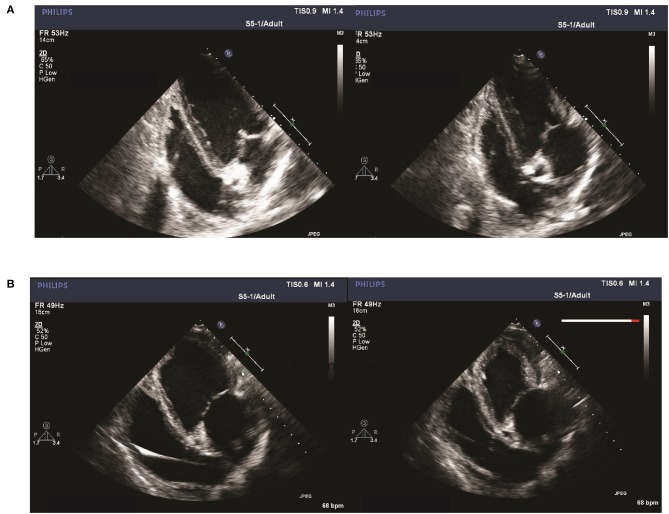
Apical 4-chamber view of left ventricular end-diastolic (left) and systolic (right) frames at follow-up of a SHAM pig **(A)** compared to an A-TP pig **(B)** obtained with the newly introduced TDE technique. Higher volumes and rounder shape of both left ventricle and atrium can be appreciated in the frame image from an A-TP pig in comparison with a control animal.

There were no significant echocardiographic differences between A-TP and SHAM pigs at the time of implantation (see Supplementary Materials). At follow-up, left ventricular systolic dysfunction was documented by a reduced LVEF (48 ± 4 vs. 68 ± 2%) and FAC (41 ± 4 vs. 63 ± 2%) in A-TP pigs compared to SHAM, respectively (Figure 6). Furthermore, A-TP induced left atrial enlargement seen by increased left atrial area (16.5 ± 1.5 vs. 11 ± 0.4 cm^2^) and left atrial end-diastolic (53 ± 7 vs. 29 ± 2 ml) and end-systolic (37 ± 6 vs. 11 ± 0.4 ml) volumes. Among left ventricular diastolic function parameters, E/A increased by 50% and the peak A decreased by 21% while other variables showed smaller changes in the same direction. E/A, MV deceleration time and E/e' ratios were not significantly different between the two groups at baseline or at follow up (Tables 1, 2). Furthermore, mitral valve regurgitation was present in eight out of nine A-TP pigs (one minimal, six mild, one moderate), as compared to only one (minimal) out of 11 in the SHAM group. These changes are consistent with the increased areas and volumes in the left atrium and left ventricle as well as with the decreased LVEF, supporting that A-TP induces signs of both diastolic and systolic dysfunction.

**Table 2:** Echocardiographic analysis of the diastolic function at follow-up.

	**SHAM follow up *n* = 12**	**A-TP follow up *n* = 9**	***P*- value**
Peak E (m/s)	0.66 ± 0.03	0.66 ± 0.05^8^	≥0.999
Peak A (m/s)	0.61 ± 0.03	0.48 ± 0.05^8^	≥0.999
Transmitral flow deceleration time (ms)	170 ± 12	156 ± 6^8^	0.625
E/A	1 ± 0.03^10^	1.52 ± 0.25^8^	0.101
e' septal (m/s)	0.08 ± 0.01	0.08 ± 0.01	≥0.999
e' lateral (m/s)	0.16 ± 0.01^8^	0.14 ± 0.02^4^	≥0.999
E/e' septal	8.5 ± 0.7	8.4 ± 0.6^8^	≥0.999
E/e' lateral	4.7 ± 0.4^8^	4.6 ± 0.6^5^	≥0.999
Tricuspid annular plane systolic excursion (mm)	20 ± 0.6^9^	20 ± 0.7^5^	0.671
LA end-diastolic volume (ml)	29 ± 2^11^	53 ± 7^8^	0.005
LA end-systolic volume (ml)	11 ± 0.4^11^	37 ± 6^8^	<0.001
LA area (cm^2^)	11 ± 0.4^11^	16.5 ± 1.5^8^	0.004
Number of pigs with mitral regurgitation (no/minimal/mild/moderate to severe)	11/1/0/0	1/1/6/1	<0.001

*Superscripts are the number of animals from which the mean in question has been calculated in case of missing observations. Values are expressed as mean ± SEM. Adjusted P values are expressed in the right column (multiple Student t-tests with Holm-Sidak's correction for multiple comparisons. Each row was analyzed individually, without assuming a consistent SD). The number of pigs with mitral regurgitation was analyzed with Fisher's exact test comparing the incidence of no, minimal or mild to severe mitral regurgitation in the two groups*.

**Figure 7:**
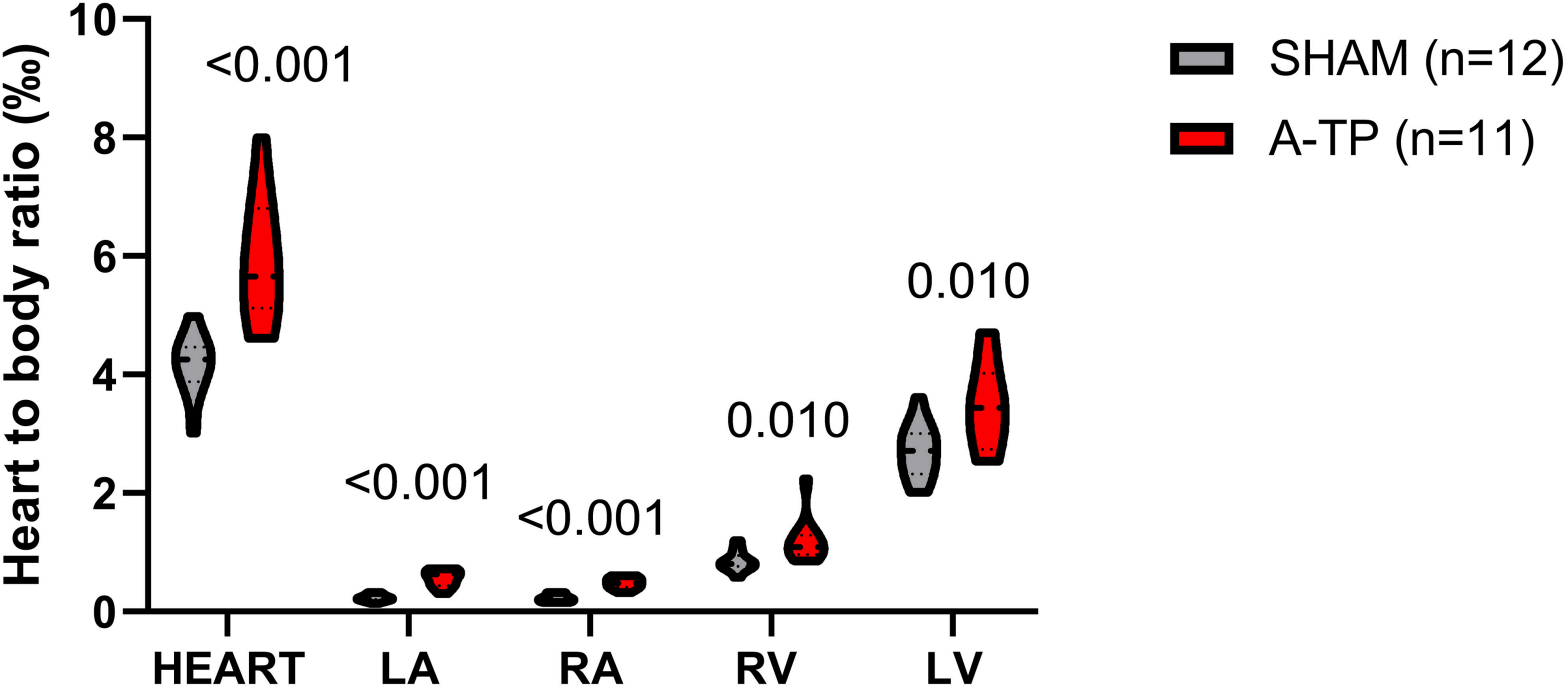
Heart-to-body weight ratio for the whole heart and for the individual chambers. The total heart-to-body weight ratio was significantly higher for the A-TP (*n* = 11) compared to the SHAM (*n* = 12) pigs (5.9 ± 0.32 vs. 4.2 ± 0.15). This was also the case for the left atrium (LA), right atrium (RA), left ventricle (LV), and right ventricle (RV) where the heart-to-body weight ratio on average was 2.3-, 2.2-, 1.4, and 1.3-fold higher in the A-TP than their SHAM counterparts, respectively.

A-TP pigs had a higher heart-to-body weight ratio than the SHAM pigs (Figure 7). This higher ratio was distributed across all four chambers.

### Analysis of Structural Remodeling and Fibrosis

To study the structural differences between A-TP and SHAM operated pigs on tissue level we performed immunohistochemical and picrosirius red stainings of the free wall of all four chambers of the heart.

#### Immunohistochemistry

**Figure 8:**
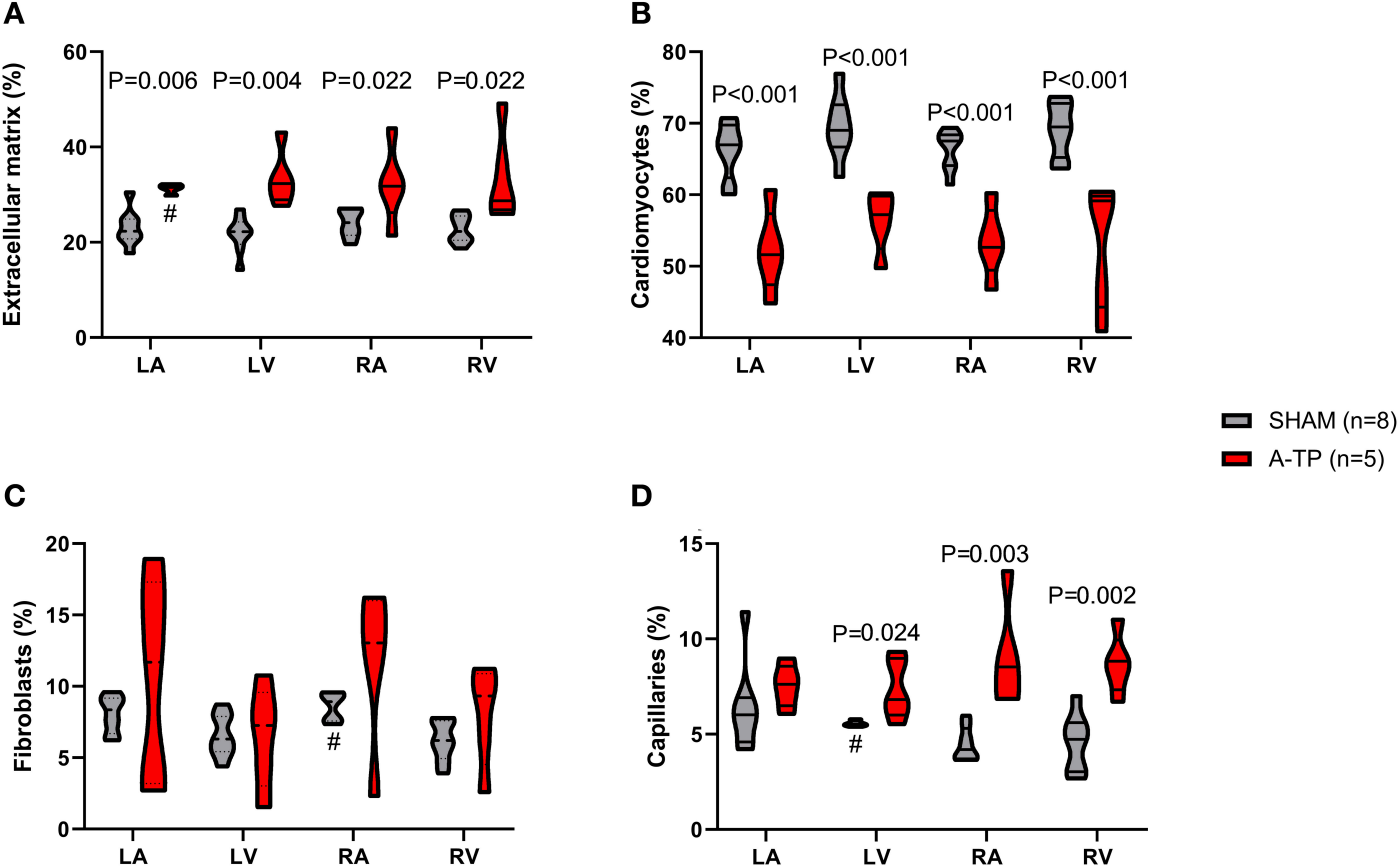
The area of ECM **(A)**, cardiomyocytes **(B)**, fibroblasts **(C)**, and capillaries **(D)** as percentage of the total tissue analyzed for each cardiac chamber. In three cases one outlier was identified and removed from the analysis—these have been marked with # in the graphs. LA, left atrium; LV, left ventricle; RA, right atrium; RV, right ventricle.

Using an automated image analyzing software (Intellesis software) the immunofluorescence stainings were quantified for the area of extracellular matrix, cardiomyocytes, fibroblasts and capillaries (Figure 5). The analysis revealed an increased area of extracellular matrix in the A-TP pigs compared to the SHAM pigs (Figure 8A). The area of extracellular matrix for A-TP (*n* = 5) and SHAM (*n* = 8) pigs, respectively, were: 31 ± 0.5 vs. 23 ± 1% in the left atrium, 33 ± 3 vs. 22 ± 1% in the left ventricle, 32 ± 4 vs. 24 ± 1% in the right atrium, and 34 ± 4 vs. 23 ± 1% in the right ventricle. Concomitantly, the area of cardiomyocytes was decreased in the A-TP pigs compared to SHAM pigs (Figure 8B). The area of fibroblasts varied quite a lot in the A-TP pig and was not significantly different from SHAM pigs (Figure 8C). The area of capillaries was increased in the A-TP pigs compared to SHAM pigs in the left ventricle (7.4 ± 0.7 vs. 5.6 ± 0.1%), right atrium (9.0 ± 1.2 vs. 4.5 ± 0.3%) and right ventricle (8.7 ± 0.7 vs. 4.6 ± 0.5%, Figure 8D). However, no significant changes were found in the area of capillaries in the left atrium between the A-TP and the SHAM group.

#### Picrosirius Red Staining for Collagen

**Figure 9:**
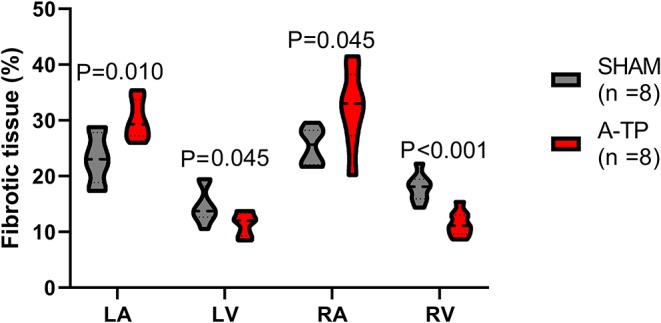
The relative area of fibrotic tissue as percentage of the total tissue area analyzed for each cardiac chamber. LA, left atrium; LV, left ventricle; RA, right atrium; RV, right ventricle.

The area of fibrotic tissue revealed by picrosirius red stainings was significantly higher in the atria of A-TP compared to SHAM pigs, 30 ± 1 vs. 23 ± 2% for the left atrium and 32 ± 2 vs. 25 ± 1% for the right atrium (Figure 9). Surprisingly, the relative area of collagen in the ventricles had decreased in the A-TP vs. SHAM pigs (11 ± 1 vs. 18 ± 1% for the right ventricle and 11 ± 1 vs. 15 ± 1% for the left ventricle). One A-TP pig was identified as an outlier and removed from the analysis.

## Discussion

By the end of the study all A-TP pigs had sustained atrial fibrillation with signs of left ventricular systolic and diastolic dysfunction, as well as atrial fibrosis and left atrial systolic and diastolic dysfunction with larger volume and area than in the SHAM pigs. The relative amount of fibrotic tissue in both ventricles was smaller in the A-TP compared to the SHAM pigs, which might imply that the higher heart weight was caused by an increase in the amount of non-fibrotic tissue. Echocardiography, collagen measurement and immunohistochemistry proved useful to show the difference in cardiac function and structural remodeling between the A-TP pigs and the SHAM pigs.

### Echocardiographic Signs of Atrial and Ventricular Dysfunction

Compared to SHAM pigs, A-TP pigs showed dysfunction in the left side of the heart seen by decreased LVEF and FAC, as well as larger left atrial volumes and areas both in systole and diastole. The latter gives an indication of volume overload and reduced contractility in the left ventricle. In the A-TP pigs, left atrial dilatation and dysfunction are present together with a significant reduction of LVEF and left ventricular fractional area change compared to the SHAM pigs. Interestingly, the left ventricular diameters in systole and diastole were not different from those in the SHAM pigs, while the left ventricular end-diastolic and end-systolic volumes both were considerably increased, implying that the left ventricle was nevertheless dilated. Clinically unrecognized mitral regurgitation has been observed to be prevalent in patients with atrial fibrillation - either as an etiologic factor leading to atrial fibrillation or developing after onset of atrial fibrillation (19). Mitral regurgitation was observed in all of the A-TP pigs and none of the SHAM pigs in the current study, lending support to the hypothesis that atrial fibrillation can cause functional mitral regurgitation (6–8), most likely due to dilatation of the mitral ring.

The very high heart rate for 43 ± 4 days in the A-TP pigs (before cardioversion) induced atrial fibrillation and started the sequence of events leading to structural remodeling and myocardial dysfunction. In fact, during the long A-TP period clinical signs of heart failure led to euthanization in 25% of 12 A-TP pigs before the follow-up evaluation. In another pig model of atrial fibrillation, 3 weeks of burst pacing with an average ventricular response rate of 270 bpm lead to clinical signs of heart failure in all the paced pigs (20). The ventricular rate control provided by digoxin (250 μg/day) in our A-TP pigs might explain why not all the A-TP pigs developed clinical signs of heart failure as was the case in the study of Bauer et al. (20). Supporting this, a study by Lin et al. (21), found that female Yorkshire-Landrace pigs, that had 25 ± 3 days of A-TP until sustained atrial fibrillation was developed, were treated with 250 μg/day of digoxin and maintained an LVEF within the normal range (21). Even though all the A-TP pigs in our study received digoxin, three pigs died from or had to be euthanized due to heart failure, supporting that our model was effective in producing both the atrial fibrillation substrate in the atria and clinically relevant ventricular remodeling.

### Signs of Structural Remodeling

The post-mortem weight of the hearts revealed that the A-TP pigs had heavier hearts than the SHAM pigs, also when looking at individual chambers.

Picrosirius red stainings showed higher relative amount of collagen in the atria and lower relative amounts in the ventricles of A-TP pigs compared to the SHAM pigs, possibly partly due to the difference between the paced atrial rate and the ventricular rate. Further, our results from the triple immunofluorescence staining revealed a larger relative amount of extracellular matrix in all chambers and a larger relative area of capillaries in the right atrium, left ventricle and right ventricle from A-TP cardiac tissue. The discrepancy in the amount of fibrosis in the ventricles from the two staining protocols could be related to the fact that picrosirius red primarily stains collagen (22), whereas WGA also binds to N-acetylglucosamin and sialic acid, that are components of glycosaminoglycans such as hyaluronic acid, which are present in the extracellular matrix. Hence, WGA stainings give a broader overview of changes in extracellular matrix content (23).

The lower percentage of collagen found in the A-TP tissue of the ventricles stained with picrosirius red may seem unexpected and seems to be caused by a higher amount connective tissue (at least proteoglycans and capillaries), even if it does not correspond to picrosirius-positive collagen.

Our findings of higher amount of fibrosis and extracellular matrix in the atria of the A-TP pig as compared to SHAM is consistent with the opinion that atrial fibrosis is an important pathophysiological factor in the development of AF [for review see (24)]. Vimentin antibodies has been validated as a staining method for fibroblasts in previous works in pigs (25). Vimentin immunofluorescence staining for fibroblasts indicated the same proportion of fibroblasts in both AT-P and SHAM pigs. The fibrosis observed, therefore does not appear to be caused by a higher number of fibroblasts in the A-TP pigs as compared to SHAM.

We speculate that the increase in extracellular matrix and fibrosis is related to atrial fibrillation induced differentiation of fibroblasts to myofibroblasts. Myofibroblasts are fibroblasts that in response to injury and pro-fibrotic stimuli differentiate and produce large amounts of extracellular matrix (24). Although vimentin was used in this study to stain fibroblasts and results showed no difference in the amount of fibroblasts between A-TP and control animals, our findings do not differ from those of Lugenbiel et al., where a 3-fold increase of α-SMA expressing cells (myofibroblasts) was found despite similar amounts of vimentin in AF and control pigs (25). According to the study of Lugenbiel, myofibroblasts are not expressing vimentin, or are expressing less vimentin compared to the α-SMA. Koga et al. recently analyzed immunophenotypes of myofibroblasts appearing in isoproterenol-induced myocardial fibrosis in rats until 28 days after injection (26). Cardiac myofibroblasts VIM+ began to appear at 8 h in the oedematous interstitial areas, and gradually increased on days 1 and 3 with a peak on day 3. However, on day 28 this had decreased to the same levels as in controls. This was also the case for the α-SMA positive cells. Based on these studies, we can speculate that fibroblasts, together with myofibroblasts, lose their vimentin expression after a long period (43 ± 3 days) of and/or, that myofibroblasts have a lower expression of vimentin in a pig model of AF lasting more than 14 days.

Isolectin B4 staining was previously applied to evaluate the angiogenesis in mouse heart (17). To the best of our knowledge, our study is the first showing a higher relative area of capillaries in the right atrium, left ventricle and right ventricle of a large animal model of atrial fibrillation induced by A-TP. A change in the same direction was found for the left atrium of A-TP pigs, but was not statistically significant. It could be speculated that in this phase of cardiac remodeling, a compensatory mechanism of neo-angiogenesis lead to augmented growth of capillaries to meet the oxygen demands of the rapid beating dilated heart.

### Study Limitations

Out of 28 A-TP pigs five died during anesthesia or had to be euthanized before the end of the study due to clinical signs of heart failure. Excluding these subjects from the study has likely biased our findings since the pigs with clinical symptoms of heart failure and the ones that died in anesthesia could be expected to have a more prominent degree of left ventricular remodeling and/or dysfunction.

Vimentin is not unique to fibroblasts since vimentin immunoreactivity has been observed in fibroblasts and vascular cells (18). The amount of fibroblasts due to inclusion of vascular cells could therefore be overestimated. Furthermore, the trained system for image analysis tends to slightly overestimate the fibrosis. However, since all samples were analyzed with the same method the results should be consistent.

In our study a staining for α-SMA was not included and it therefore remains speculation that ECM/fibrosis is related to an increased number of myofibroblasts.

The anesthetic regimen used in the study may have influenced hemodynamic parameters, however the same protocol has been used for all pigs which should ensure consistency.

The sample sized in our groups are small and the study is therefore only powered to find relatively large and uniform changes.

Since the sample sizes are small in our groups, we used only females to keep the groups as homogenous as possible at baseline in order to minimize the standard deviation. It is, however, a limitation that only one sex was used in this study.

## Conclusion

In this pig model, A-TP-induced atrial fibrillation was associated with echocardiographic signs of atrial and ventricular systolic and diastolic dysfunction, as well as signs of structural remodeling characterized by an increased relative abundance of extracellular matrix. However, the addition of digoxin as a rate controlling agent reduced the effects on the left ventricle. This study confirmed that atrial fibrillation alone can lead to structural remodeling and atrial and ventricular dysfunction. Echocardiography provided a useful tool to detect atrial fibrillation induced changes that correlated well with macroscopic and histological findings.

## Data Availability Statement

The datasets generated for this study are available on request to the corresponding author.

## Ethics Statement

The animal study was reviewed and approved by Danish Ministry of Environment and Food.

## Author Contributions

JD, BB, MG, JK, and CC contributed conception and design of the study. SS, LO, NE, and CC performed and/or helped with analysis of echocardiographic data. MF, FG, and AZ participated in results elaboration. JD and CC wrote first draft of manuscript. All authors contributed to manuscript revision, read, and approved the submitted version.

### Conflict of Interest

JD, MG, NE, and BB are fully or partly employed by Acesion Pharma. The remaining authors declare that the research was conducted in the absence of any commercial or financial relationships that could be construed as a potential conflict of interest.

## Abbreviations

A-TP: atrially tachypaced
FAC: fractional area change
FS: fraction shortening
IVSd: intraventricular septum in diastole
IVSs: intraventricular septum in systole
LVEF: left ventricular ejection fraction
LVIDd: left ventricular internal diameter in diastole
LVIDs: left ventricular internal diameter in systole
MV: mitral valve
TAPSE: Tricuspid annular plane systolic excursion
TTE: transthoracic echocardiography.
