# Supplementary material for: Characterization of Atrial and Ventricular Structural Remodeling in a Porcine Model of Atrial Fibrillation Induced by Atrial Tachypacing
Source: Front Vet Sci. 2020 Apr 9;7:179. doi: 10.3389/fvets.2020.00179 (PMC7160334; doi:10.3389/fvets.2020.00179)
Supplement: Supplementary file 1 [file Data_Sheet_1.docx]

Supplementary Materials

## Substernal surgical procedure for trans-diaphragmatic echo.

A 10 cm mid-line incision starting at the sternal manubrium cartilage was performed followed by blunt dissection of sub-cutis and abdominal muscles. The abdominal peritoneum was left intact. Access towards the diaphragm was done using soft tissue scissors for blunt dissection (Metzenbaum) and by fingers. The cavity towards the diaphragm was filled with echocardiographic water-based gel and the probe was placed on the diaphragmal region of the cardiac apex (also see figure 2C).

## Trained model for image segmentation.

Every segmentation step was done by the deep learning algorithms and by the Python infrastructure of the software module Zen Intellesis. Zen Intellesis (Zeiss) is a custom-made program. It extracts 33 selected features and the neural network classifies automatically according to these features. The user provides several rounds of training annotation in order to indicate to the pixel classifiers what the ground truth is. The original validation of the software can be found in these two papers (1,2).

## Tables with equipment, drugs, and software

Table 1: Equipment

| **Equipment** | **Producer** | **Model** | **Rationale for choosing this** |
| --- | --- | --- | --- |
| AF Neurostimulator | Medtronic, Dublin, Ireland | Synergy versitrel | Implantable stimulator capable of delivering sufficiently fast and long electrical impulses for cardiac tachypacing. |
| AF Neurostimulator | Medtronic, Dublin, Ireland | Itrel 3 | Implantable stimulator capable of delivering sufficiently fast and long electrical impulses for cardiac tachypacing. Slightly smaller than the synergy versitrel making it easier to implant. No longer in production. |
| Sterile filter | Thermo Scientific, Waltham, Massachusetts, USA | Nalgene, Rapid flow 90 mm filter unit, 250-500 ml. | State of the art product for sterile filtration. |
| Implantable leads | St Jude Medical, Little Canada, Minnesota, US | Tendril (2088T/58 cm) | State of the art electrical lead with active fixation. Used routinely for patients. |
| Televet, Holter monitor | Engel Engineering Service GmbH, Heusenstamm, Germany | Televet-100 | State of the art Telemetric ECG System for veterinary medicine. |
| Infusion pump | Shenzhen Shenke Medical Instrument Technical Development Co.,Ltd., Shenzhen P.R.China | SK-500I syringe pump | Syringe pump capable of delivering up to 500 ml/h. |
| 50 ml syringe | Medical Surgical Systems BD, Albertslund, Denmark | BD Plastipak 50 ml Luer-Lok syringe | Standard 50 ml Luer-Lok syringe compatible with most syringe pumps. |
| Patient Monitor | Agilent, Glostrup, Denmark, | Viridia | Monitoring of ECG, Blood pressure SP0_2_ and temperature of the pig during surgery. |
| Respirator | Demeca, Rødovre, Denmark | Siesta i TS | Respiration of the pig during surgery. 500 mL/min 0_2_ and 2500 mL/min air. |
| Infusion pump | B. Braun Melsungen AG,  Melsungen, Germany | Infusomat space | Pump for infusion of Propofol. |
| Infusion pump | B. Braun Melsungen AG, Melsungen, Germany | Perfuser compact | Syringe pump for infusion of fentanyl. |
| Infusion pump | Heska,  Loveland, Colorado, USA | Vet/IV | Infusion pump for saline solution. |
| Cardiovascular ultrasound machine | (Philips Health care, Amsterdam, The Netherlands) | iE33 machine equipped with an S5-1 transducer (3.5 MHz) | Ultrasound machine for echocardiographic images acquisition |

Table 2: Drugs

| **Drug** | **Producer** | **Strength** | **Dosing** | **Rationale** |
| --- | --- | --- | --- | --- |
| Zoletil pig mix | Unit for experimental medicine, Copenhagen University | Solution for injection, 250 mg dry tiletamin+zolazepam, 6.5 ml xylazine 20 mg/ml, 1.25 ml ketamine 100 mg/ml, 2.5 ml butorphanol 10 mg/ml, and 2 ml methadone 10 mg/ml | 0.1 ml/kg, IM | Used for pre-anaesthesia of the pigs. |
| Gentacoll® patches | Swedish Orphan Biovitrum | 32.5 mg gentamycin in 5x5 cm patch | 32.5 mg in implantation pocket. | Broad-spectrum antibiotic to avoid infection in the implantation pocket. |
| Propolipid | Fresenius Kabi | Liquid for infusion, emulsion of 10 mg/ml propofol. | 15 mg/kg/h, IV | Anaesthetic agent |
| Fentanyl | “B. Braun”,  “2Care4”, or “Sandoz” | Solution for injection, 50 µg/ml | 5 µg/kg/h | Opioid. Anaesthetic and analgesic agent. |
| Norostrep Vet. | ScanVet Animal Health A/S, Fredensborg | Liquid for infusion, emulsion of 200.000 IU (200 mg)/ml. | 2 ml on the pacemaker just before implantation. | Antibiotic for penicillin- and dihydrostreptomycin-vulnerable bacteria. |
| Curamox prolongatum Vet. | Boehringer Ingelheim | Liquid for infusion, emulsion of 150mg amoxicillin/ml | 15 mg/kg, IM | Broad-spectrum antibiotic for amoxicillin-vulnerable bacteria with prolonged effect (>48 h) |
| Metacam | Boehringer Ingelheim | 15 mg meloxicam/ml | Oral suspension  0.5 mg/kg PO daily for three days after implantation | NSAID for post operational pain. |
| Clamoxyl Vet. | Orion Pharma Animal Health A/S | 510 mg amoxicillin/g | Powder for oral solution 40 mg/kg PO daily for 5 days after implantation | Broad-spectrum antibiotic for amoxicillin-vulnerable bacteria. |

All drugs used in this study are commercially available and sourced from the open market within the European Union. The zoletil pig mix is not a marketed combination and was prepared by the Unit for Experimental Medicine at Copenhagen University.

Table 3: Software

| **Software** | **Supplier** | **Rationale** |
| --- | --- | --- |
| GraphPad Prism 8.2.0 | GraphPad Software, Inc. | Software for simple statistical analyses and for graphical representations of data. |
| LabChart 7.3.7. | ADInstruments | A platform for multiple recording devices to work together, allowing the acquisition of biological signals from multiple sources simultaneously. Allows for semi-automated analyses of ECGs. |
| Televet ECG software 6.0.0 | Engel Engineering Services GmbH, Germany | Works with the Televet100 Holter monitor for acquisition of ECGs from conscious pigs. |
| EchoPAC software | GE healthcare, USA | Software for echocardiographic images analyses |

## Post-operative treatment

Postoperative analgesia was accomplished by administration of Meloxicam (Metacam, Boehringer Ingelheim, Germany) 0.5 mg/kg orally (PO) once daily for post-operatory pain. Amoxicillin (Clamoxyl vet 510 mg/g, Orion Pharma Animal Health A/S, Copenhagen, DK) , powder for oral solution, 40 mg/kg PO daily was given for 5 days after implantation to lower the risk of postoperative infections.

Table 4: Cardioplegic solution

| **Final Solution** | **Total Volume – 2L** |
| --- | --- |
| 20x Stock | 100mL |
| NaHCO_3_ | 10mL |
| MiliQ H_2_O | Up to 2000ml |

Table 5: Cardioplegic stock solution (x20)

| **Stock Solution** | **Total Volume – 2L** |
| --- | --- |
| KCl (2M) | 320mL (320mM) |
| NaCl (4M) | 1100mL (2200mM) |
| CaCl_2_ (1M) | 48mL (24mM) |
| MgCl_2_ (0,1M) | 640mL (320mM) |
| MiliQ H_2_O | Up to 2000ml |

**Table 6: overview of detailed inclusion/exclusion of the pig population in the procedures described in the manuscript**

| Pig ID | **Experimental group** | **Completed the study** | **Echo at implant** | **Echo at follow-up** | **Heart Weight/BW** | **Immunohistochemistry** | **Picrosirius red staining** |
| --- | --- | --- | --- | --- | --- | --- | --- |
| 274 | A-TP | NO | NO | NO | NO | NO | NO |
| 275 | A-TP | YES | YES | YES | YES | NO | YES |
| 281 | A-TP | NO | NO | NO | YES | NO | YES |
| 105 | A-TP | NO | NO | NO | NO | NO | NO |
| 319 | A-TP | NO | NO | NO | NO | NO | NO |
| 381 | A-TP | YES | NO | NO | YES | YES | YES |
| 382 | A-TP | YES | YES | YES | YES | YES | YES |
| 283 | A-TP | YES | NO | YES | YES | NO | YES |
| 303 | A-TP | YES | YES | YES | YES | YES | YES |
| 304 | A-TP | YES | YES | YES | YES | NO | YES |
| 380 | A-TP | YES | YES | YES | YES | YES | YES |
| 406 | A-TP | YES | YES | YES | YES | YES | YES |
| 146 | A-TP | YES | YES | YES | YES | NO | NO |
| 147 | A-TP | YES | YES | YES | YES | NO | NO |
| 2 | SHAM | YES | NO | YES | YES | YES | YES |
| 8 | SHAM | YES | NO | YES | YES | YES | YES |
| 407 | SHAM | YES | NO | NO | NO | NO | NO |
| 1(17) | SHAM | NO | NO | NO | NO | NO | NO |
| 141 | SHAM | YES | YES | YES | YES | YES | YES |
| 142 | SHAM | YES | YES | YES | YES | YES | YES |
| 153 | SHAM | YES | YES | YES | YES | YES | YES |
| 189 | SHAM | YES | YES | YES | YES | YES | YES |
| 190 | SHAM | YES | YES | YES | YES | YES | YES |
| 191 | SHAM | YES | YES | YES | YES | YES | YES |
| 1(18) | SHAM | YES | NO | YES | YES | NO | NO |
| 33 | SHAM | YES | NO | YES | YES | NO | NO |
| 34 | SHAM | YES | NO | YES | YES | NO | NO |
| 99 | SHAM | YES | YES | YES | YES | NO | NO |
| Number of pigs (n) | 14 A-TP  14 SHAM | 10 A-TP  13 SHAM | 8 A-TP  7 SHAM | 9 A-TP  13 SHAM | 11 A-TP  12 SHAM | 5 A-TP  8 SHAM | 9 A-TP  8 SHAM |

Pigs marked in red did not complete the study and were not included in the manuscript. Pig #407 marked in yellow did complete the study but was not included in the statistical analyses. Pigs marked in light blue were either euthanized due to the presence of congestive cardio-respiratory clinical signs at the end of the study period (#281) either died during anaesthesia at the follow-up experiment (#381 and #382). Nevertheless, these pigs were included in the statistical analyses. NO = not included; YES = included; BW = body weight.

**Table 7: Systolic and diastolic myocardial function at baseline.**

|  | SHAM  at implantation  *n* = 7 | A-TP  at implantation  *n* = 8 | P values |
| --- | --- | --- | --- |
| LVIDd (mm) | 37.7 ± 2.5 | 36 ± 1.6 | ≥0.999 |
| LVIDs (mm) | 23.6 ± 1.7 | 22.1 ± 1.1 | ≥0.999 |
| IVSd (mm) | 6.9 ± 0.6 | 6.1 ± 0.3 | 0.998 |
| IVSs (mm) | 8.9 ± 1 | 8.6 ± 0.6 | ≥0.999 |
| FS (%) | 37.3 ± 1.5 | 43 ± 2 | 0.621 |
| LV end-diast. Vol. (ml) | 51.6 ± 4.9 | 51.3 ± 5.4 ^7^ | ≥0.999 |
| LV end-syst. Vol. (ml) | 17.9 ± 2.5 | 17.4 ± 3 ^7^ | ≥0.999 |
| EF (%) | 66.4 ± 2.3 | 66.3 ± 2 ^7^ | ≥0.999 |
| LV end-diast. Area (cm^2^) | 10.8 ± 0.53 | 11.91 ± 0.72 ^7^ | 0.995 |
| LV end-syst. Area (cm^2^) | 4.5 ± 0.3 | 4.6 ± 0.3 ^7^ | ≥0.999 |
| LVFAC (%) | 57.9 ± 3 | 61.6 ± 2 ^7^ | ≥0.999 |
| LA end-diast. Vol. (ml) | 11.3 ± 1.3 ^6^ | 12.7 ± 0.7 ^7^ | ≥0.999 |
| LA end-syst. Vol. (ml) | 5.3 ± 1.6 ^6^ | 5.5 ± 0.5 ^7^ | ≥0.999 |
| LA area (cm^2^) | 6 ± 0.6 ^6^ | 6.2 ± 0.4 ^7^ | ≥0.999 |
| MaxAoFlow (cm/s) | 0.97 ± 0.11 | 0.91 ± 0.07 | ≥0.999 |
| MR, n | 7/0/0/0 | 8/0/0/0 | ≥0.999 |
| Peak E (m/s) | 0.7 ± 0.05 ^6^ | 0.7 ± 0.04 ^8^ | ≥0.999 |
| Peak A (m/s) | 0.48 ± 0.07 ^6^ | 0.56 ± 0.07 ^8^ | ≥0.999 |
| EDT (ms) | 144 ± 6.4 ^6^ | 152 ± 13.5 ^8^ | ≥0.999 |
| E/A | 1.52 ± 0.11 ^6^ | 1.36 ± 0.13 ^8^ | ≥0.999 |
| e' septal (m/s) | 0.07 ± 0.01 | 0.09 ± 0.01 ^7^ | 0.447 |
| e' lateral (m/s) | 0.14 ± 0.01 | 0.14 ± 0.02 ^5^ | ≥0.999 |
| E/e' septal | 10.65 ± 0.94 ^6^ | 8.54 ± 0.96 ^7^ | 0.970 |
| E/e' lateral | 4.85 ± 0.33 ^6^ | 4.94 ± 0.42 ^5^ | ≥0.999 |
| TAPSE (mm) | 20 ± 0.9 ^5^ | 20.4 ± 1 ^7^ | ≥0.999 |

LVIDd, Left ventricular internal diameter in diastole; LVIDs, Left ventricular internal diameter in systole measured with M-mode in the right parasternal short-axis view; Volumes of LA and LV and LA area measured with SMOD method in left apical 4 chamber view; LV area in systole and diastole measured in short-axis view using 2D echocardiography. MV Peak E, early diastolic velocity; MV Peak A, atrial contraction velocity; EDT, transmitral flow deceleration time; E:A, Peak E and A ratio; E' (septal and lateral), isovolumic relaxation time; E/E'(septal and lateral), peak E and isovolumic relaxation time ratio; TAPSE, tricuspid annular plane systolic excursion; MR, n, Number of pigs with mitral regurgitation (no/minimal/mild/moderate to severe).Superscripts are the number of animals from which the median in question has been calculated in case of missing observations. Values are expressed in mean ± SEM. Adjusted P values are expressed in the right column (multiple Student t-tests with Benjamini, Krieger and Yekutieli’s correction for multiple comparisons, Mann-Whitney test for body weight and heart rate).

1. Andrew M, Hornberger B. Benchmarking of Machine Learning and Conventional Image Segmentation Techniques on 3D X-ray Microscopy Data. Microsc Microanal. 2018 Aug;24(S2):120–1.

2. Volkenandt T, Freitag S, Rauscher M. Machine Learning Powered Image Segmentation. Microsc Microanal. 2018 Aug;24(S1):520–1.
